# The effect of vitamin D supplementation on body composition in nursing mothers with overweight or obesity: a randomized double-blind placebo-controlled clinical trial

**DOI:** 10.1186/s40795-022-00664-y

**Published:** 2023-01-02

**Authors:** Zohre Gerveieeha, Fereydoun Siassi, Mostafa Qorbani, Rezgar Shahi Menbar, Mohammad Hossein Ahmadi, Gity Sotoudeh

**Affiliations:** 1 Department of Community Nutrition, School of Nutritional Sciences and Dietetics, Tehran University of Medical Sciences, Hojatdost street, Naderi street, Keshavarz Blvd, Tehran, Iran; 2 Non-communicable Diseases Research Center, Alborz University of Medical Sciences, Karaj, Iran; 3 Department of Community Nutrition, School of Nutritional Sciences and Dietetics, Isfahan University of Medical Sciences, Isfahan, Iran; 4 Department of Medical Laboratory Sciences, school of Allied Medicine, Qazvin University of Medical Sciences, Qazvin, Iran

**Keywords:** Nursing mothers, Body composition, Overweight, Obesity, Vitamin D, Supplementation, 25(OH) D: 25-Hydroxy vitamin D.

## Abstract

**Background:**

This study aimed to evaluate the effect of vitamin D3 supplementation on body composition and anthropometric measures of nursing mothers.

**Methods:**

In a double-blind, randomized clinical trial, 90 nursing mothers with overweight or obesity were randomized into three groups for 12 weeks: two groups of vitamin D3 supplementation (2000 IU/d (VD1), *n* = 32 and 4000 IU/d (VD2), *n* = 29) and placebo (PL) group (*n* = 29). The information on body composition was obtained using the body impedance analysis (BIA) method. Serum 25-Hydroxy vitamin D (25(OH) D), Intact Parathyroid Hormone (iPTH), calcium, and phosphorus were measured before and after the intervention. Data were analyzed based on the intention-to-treat (ITT) method. Two-way repeated measure ANOVA (mixed ANOVA) was applied to assess whether the mean changes in the results from baseline to 12 weeks differ in the three groups.

**Results:**

There was a significant increase in the serum 25(OH) D concentration in the VD2 group compared to VD1 and PL groups (mean change (MC), 12.3 ng/ml; 95% CI, 9.4/15.0, *p*-value < 0.001). In addition, fat mass (MC, − 4.3 kg; 95% CI, − 7.0/− 1.1, *p*-value < 0.007), fat mass index (MC, − 1.6; 95% CI, − 2.6/− 0.5, *p*-value < 0.006) and body fat percentage (MC, − 8.1; 95% CI, − 12.0/− 4.2, *p*-value < 0.007) reduced in VD2 group as compared with VD1 and PL groups.

**Conclusion:**

The intake of 4000 IU/d vitamins D3 supplementation would elevate circulating 25(OH) D concentrations in nursing mothers with overweight or obesity and improve some indices of body composition.

**Trial registration:**

Iranian Registry of Clinical Trials (http://www.irct.ir: IRCT20140413017254N6) registered on 11-04-2018.

**Graphical Abstract:**

The graphical abstract of this clinical trial, is a figure that explains the final results of the manuscript in a clear and attractive way
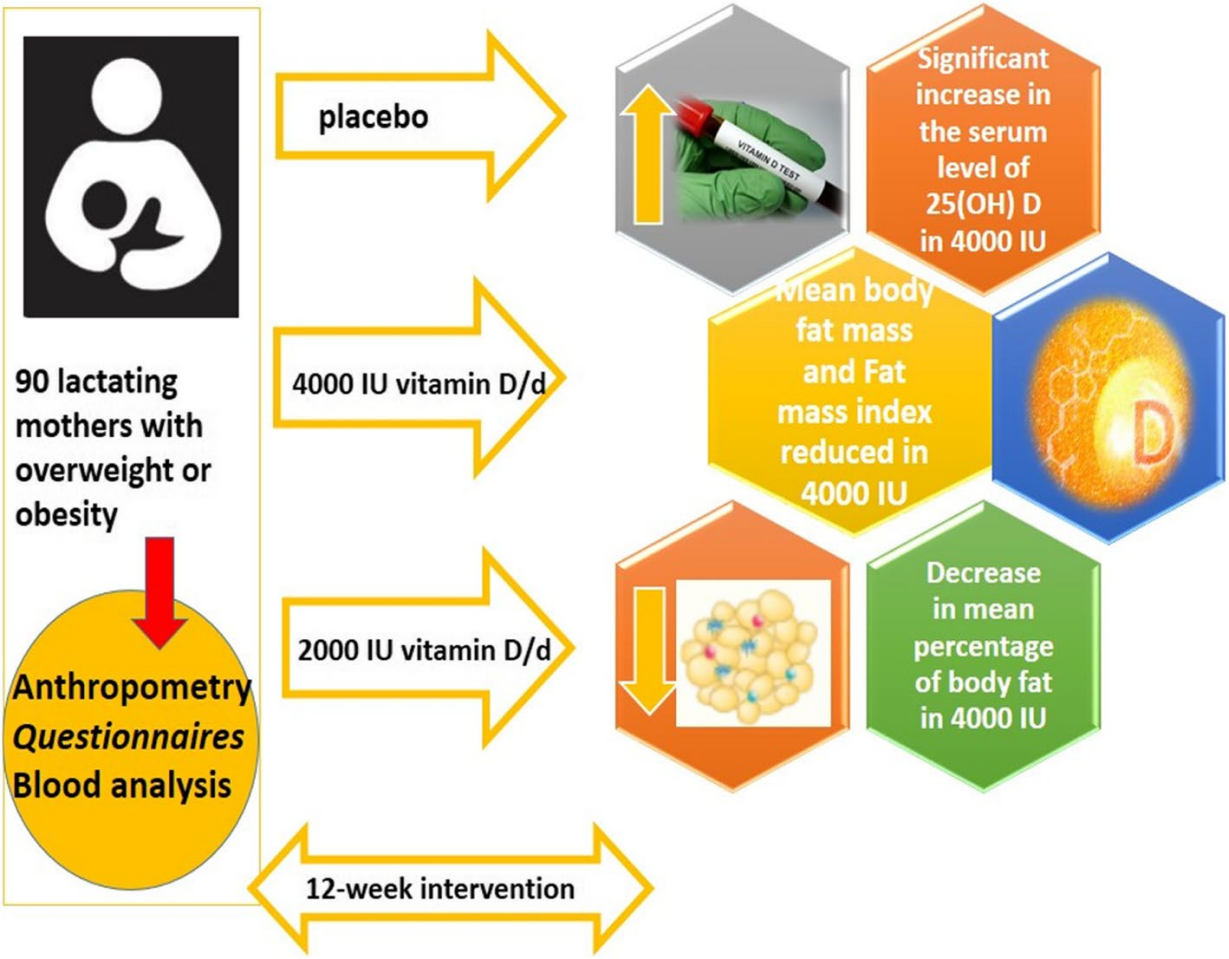

## Background

Nearly half of the nursing mothers in developed countries suffer from overweight or obesity during lactation [1]. This figure in Iran was reported as 31.7–37.3% [2, 3]. Factors such as unhealthy dietary intake, low physical activity [4], higher serum leptin concentration [5] or pre-pregnancy high body mass index (BMI) [6], and low serum 25(OH) D concentration [7] (< 20 ng/ml (50 nmol/l) [8–10]) may cause women to gain more weight during pregnancy, which may lead to obesity in lactation duration [11].

The global prevalence of a vitamin D deficiency in pregnant and nursing women was reported to be 21–85% [12]. In Iran, the Second National Survey on the status of micronutrients showed that 85% of pregnant women are exposed to vitamin D deficiency (≤ 30 ng/ml) or a severe deficiency (≤10 ng/ml) Therefore, a vitamin D deficiency may be high in nursing mothers too [13], which may be due to less frequent multivitamin intake during lactation as compared with pregnancy [14]. Other causes, such as an increase in the body’s need for bone mass [15], lack of adequate sunlight exposure [16], and a low intake of dietary vitamin D [12], can contribute to vitamin D deficiency [17, 18].

The concentration of serum 25(OH) D has an inverse association with body weight, BMI, and fat mass (FM) [19, 20]. After exposure to sunlight, the increase in serum concentrations of 25(OH) D was 43% less in obese individuals than non-obese participants [21]. However, the findings of a study reported that volumetric dilution causes a difference in serum 25(OH) D concentrations between non-obese and obese women, leading to vitamin D deficiency [22, 23].

Vitamin D supplementation may affect FM and body weight in overweight people [8, 9]. A systematic review reported that the effect of vitamin D supplementation on weight loss in participants with obesity was not conclusive [8, 9]. However, some of the interventions included in this review, investigated the combination therapy of vitamin D with other treatments such as energy restriction, calcium, or omega-3 supplements [8, 9]. Thus, the interpretation of the findings remains controversial. A meta-analysis provided evidence of an inverse association between FM and serum 25(OH) D. However. It does not support the hypothesis that vitamin D supplementation can augment body-fat loss [24]. The impact of different doses of vitamin D supplements on the body composition in nursing women with overweight or obese is unknown. Only one trial has previously investigated the impact of vitamin D supplementation during lactation on mothers with different weight status [25]. However, data on nursing mothers with obesity have not been reported separately. This study showed significant negative correlations between serum 25(OH) D and FM [25]. Therefore, more studies were needed to determine whether higher vitamin D intake might reduce body fat in nursing mothers. We hypothesized that the higher dose of vitamin D supplementation might improve serum 25(OH) D concentration and body composition. The present study aimed to investigate the effects of larger doses of vitamin D3 supplementation on serum 25(OH) D concentration and body composition in nursing mothers with overweight or obesity.

## Methods

### Participants and study design

The present study is part of a double-blind, randomized clinical trial, which aimed to investigate the effects of 2000 and 4000 IU/d of vitamin D3 supplementation on serum 25(OH) D concentration, body composition, and anthropometric measures in nursing mothers with overweight or obesity, as well as to assess the growth and risk of infection in their infants. Postpartum nursing mothers were recruited at the private hospital’s maternity ward in Qazvin province from November 2018 to March 2019 (autumn to winter). Inclusion criteria were participants aged 20–49 with a BMI 25–39.9 kg/m^2^ who delivered at term (gestational age of 37–42 weeks),and a birth weight appropriate for gestational age (2500–3900 kg). In addition, the mothers had to undertake to continue breastfeeding for the 3 months of the study. The exclusion criteria were having diagnosed gastrointestinal disorders interfering with bowel function, having severe hepatic, renal, inflammatory, cancer, diabetes, hypertension, epilepsy, and thyroid diseases, or taking any medication, a history of smoking or alcohol consumption in the past month and adhering to a specific diet during the past 12 weeks.

The sample size was calculated according to Roosta et al.’s study [26] by two mean comparison formulas, type I error (α) =0.05, type II error (β) =0.2, and mean (Standard Deviation) of waist circumference (WC) changes. Mean changes of WC after supplementation of vitamin D at the end of the study of Roosta et al. [26] were equal to 1.91 ± 1.7 cm and 0.55 ± 1.04 cm in the intervention and control groups, respectively. For power = 0.8 and (α) value = 0.05, the sample size was calculated to equal 18. Since there were 3 groups in this study, the calculated sample size was multiplied by √ (the number of groups) [27]. Finally, the required sample size for this study was 90 participants.

The protocol for the present study was published previously [28] and was registered in the Iranian Registry of Clinical Trials (http://www.irct.ir: IRCT20140413017254N6) on 11-04-2018. The Ethics Committee of Tehran University of Medical Sciences approved the study (IR.TUMS REC 1397429).

### Outcomes

The outcomes of the study were WC, weight, BMI, body fat percentage (BFP), FM, fat-free mass (FFM), skeletal muscle mass, relative fat mass index (RFMI), fat mass index (FMI), serum concentrations of 25(OH) D, calcium, iPTH and phosphorus.

### Randomization and intervention

Ninety nursing women with overweight or obesity were randomly allocated to three groups: two groups of vitamin D3 supplementation 2000 IU/d (VD1, *n* = 32), 4000 IU/d (VD2, *n* = 29) and placebo group (PL, *n* = 29). The participants were randomly assigned to groups with a 1:1:1 randomization ratio. Randomization was performed by an assistant using permuted block randomization method, and stratified randomization was employed to match the women based on age (20–34 and 35–49 years) and BMI (25–29.9 and 30–39.9 kg/m^2^). The intervention allocation was blinded for both investigators and participants. Vitamin D3 (cholecalciferol) and placebo (lactose) supplements were in the form of nano microcapsules, which have provided by the Nano Hayat Darou Industrial Co. (Tehran, Iran). Mothers are instructed to consume two nano micro capsules daily, one capsule with lunch and dinner. The capsules were identical in size, color, and shape. The vitamin D3 supplement and placebo were packed by the Pharmacy. The double-dummy method was used for the double-blind study. The intervention started approximately 3 days after delivery and continued up to 12 weeks. Mothers were called up every week. The number of returned capsules were recorded at the final visit to calculate compliance and adherence to the intervention.

### Socio-demographic, sunlight exposure, physical activity measurement and dietary analysis

Questionnaire inquiries on socio-demographic data were completed by participants at the beginning of the study. The researcher asked sunlight exposures and physical activity at the beginning and the end of the study. Duration of outdoor activity and sunlight avoidance histories such as usage of umbrella and sunscreen were asked. Participants were also inquired about wearing long or short sleeves clothes, short or long pants and the traditional Islamic veil. This questionnaire was obtained from a previous study, which investigated the validity and reliability of the questionnaire [29]. The reliability of the questionnaire was calculated as a pilot and through (Test re Test) for 20 students. The coefficient of stability was obtained as 0.85 [29]. The validity of the questionnaire was determined by the opinion of ten faculty members of the Kurdistan University of Medical Sciences.

The short form of the International Physical Activity Questionnaire (IPAQ) was used to assess physical activity patterns during the previous week (http://www.ipaq.ki.se 2017). The questionnaire consists of seven questions to evaluate the intensity of activities. Scores less than 600 metabolic equivalents (MET) minutes per week show low activity, scores expressed in 600–3000 MET minutes per week show moderate physical activity, and scores more than 3000 MET minutes per week represent intense physical activity.

For the assessment of dietary intakes, two 24-hour food recalls were completed at the baseline and end of the study. One of the 24 -hour recalls was filled for one of the working days of a week and another one on holidays. The Nutritionist IV software version 4.1 (First Databank Division, the Hearst Corporation, and San Bruno, CA) was used to estimate dietary intake of nutrients.

### Anthropometry and body composition assessment

Height was measured using a fixed stature meter (Model No.26 SM). The BMI was calculated as weight in kilograms divided by the square of the height in meters. Bioelectrical Impedance Analysis (BIA) was performed using In Body model 270 as a body composition analyzer (In Body Co., Ltd. Seoul, Korea), to assess weight, FM (kg), FFM (kg), skeletal muscle mass (kg), BFP (%), FMI as FM (kilogram) divided by the square of height (meter) [30], and RFMI for women calculated as 76 − (20 height × WC in meters) [31]. All measurements were done at 8–10 a.m.

### Biochemical assay

After an 8-h overnight fasting, the registered staff nurses took the venous blood samples between 9 and 10 a.m. Then blood samples were centrifuged at 3000 rpm for 10 min at 4 °C to obtain the serum and were stored at − 21 °C until biochemical analyses. Serum was used for analyzing calcium, phosphorus, iPTH, and 25(OH) D concentrations at the baseline and end of the study. Assay performance was measured using the kit, and standard laboratory procedures, and performance were within acceptable limits. Calcium and phosphorus concentrations were measured by colorimetric enzymatic test (Pars Azmun Co., Tehran, Iran) by photometric UV test BILT1500. 25(OH) D and iPTH were measured by Enzyme-linked Immune Sorbent Assay (ELISA) (Monobind, Inc. Lake Forest, CA (92630), the USA) and (Biomerica, Inc. Irvine, CA (92614) USA), respectively. The inter- and intra-assay coefficients of variation (CV) for calcium, phosphorus, 25(OH) D, and iPTH were 1.04 and 2.01%, 1.61 and 2.22%, 3.87 and 4.55%, 4.5, and 3.9%, respectively.

### Statistical analyses

All statistical analyses were performed by SPSS version 24 (SPSS Inc., Chicago, IL, USA). Analyses were done based on ITT analyses. The ITT population consisted of all the enrolled and randomized participants. In the ITT method, information on the baseline was considered a covariate. Multiple imputation methods were used to impute missing values. The missing data were imputed using a linear regression imputation method. The BMI and serum 25(OH) D concentrations in each study group were used for multiple imputations. The normal distribution of variables was tested and confirmed by Kolmogorov–Smirnov test. The baseline measurements and dietary intakes of participants in the three groups were compared using one-way analysis of variance (ANOVA) test for quantitative variables with normal distribution, the Kruskal Willis test for non-parametric, and the Chi-square test for qualitative variables. The differences between before and after values of body composition measures were computed; then, the independent t-student test was used to determine the relationship between the mean changes of body composition measures and serum 25(OH) D status.

Two-way repeated measure ANOVA (mixed ANOVA) with Bonferroni correction was applied to assess the time effect and time-by-treatment (two doses of vitamin D or placebo) interaction effect on all outcome measures. The models were adjusted for saturated fatty acids (SFA) intake that was different between treatment groups at the baseline with a *p*-value < 0.05. Since weight loss during lactation might complicate the findings relating to vitamin D supplementation, the body composition variables (except for weight and body mass index) were also adjusted for mean change of weight. *P* < 0.05 was considered statistically significant.

## Results

Compliance was high, and more than 82, 93, and 89% of the capsules were consumed in VD1, VD2, and PL groups.

In the early days of the intervention, two women from the VD1 group complained of flatulence and diarrhea. However, no side effects were reported in VD2 and PL groups. The serum calcium concentrations in all three treatment groups were in the normal range (8.6–10.6 mmol/l) before and after treatment.

### Characteristics of participants and dietary intake

**Fig. 1:**
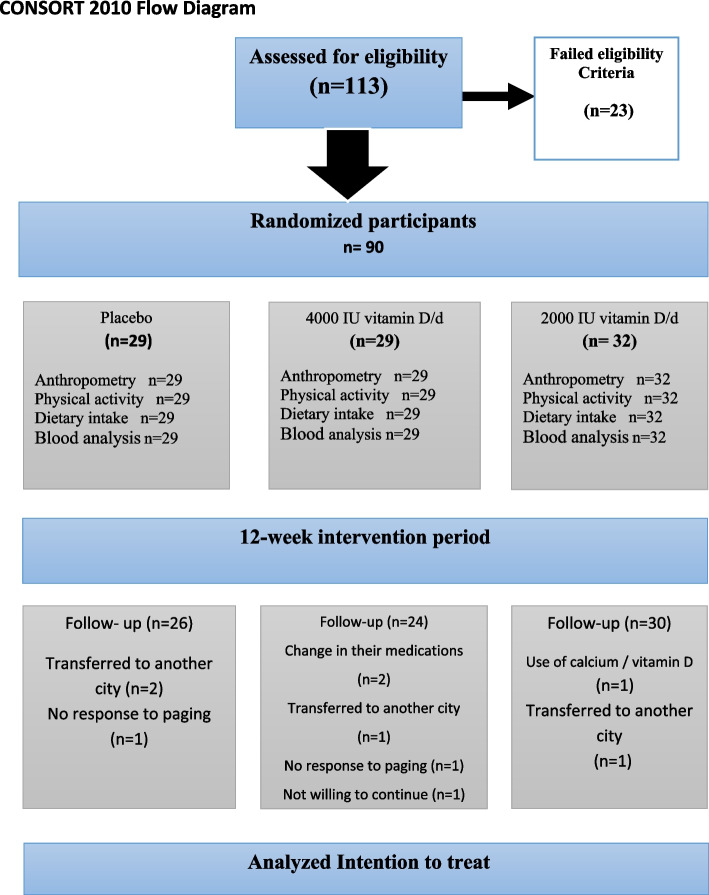
Participant’s flow diagram

Ten out of ninety participants did not complete the study for different reasons (two in the VD1 group, five in the VD2 group, and three in the PL group). One of them is taking calcium/vitamin D supplements due to a lumbar disc, two of them changed their medications. Four women could not continue the study due to the change in their husband’s working conditions, and one woman refused to take supplements at the beginning of the intervention after blood sampling, and two mothers did not respond to paging (Fig. 1).

There were no significant differences between the three groups in terms of general characteristics at the baseline and the end of the study (*p*-value> 0.05). All the participants took iron supplement after delivery. There were no significant differences regarding iron intakes between groups (*p*-value> 0.05) (Table 1). In addition, There were no significant differences among the three groups in terms of the intake of energy and nutrients, except for the intake of SFA, which was significantly higher in the PL group compared with the other two groups at the baseline of the study (*p*-value = 0.02) (Table 2).

**Table 1:** General characteristics of nursing mothers at the baseline of study

Variable		PL^a^ (*n* = 29)	VD1^a^ (*n* = 32)	VD2^a^ (*n* = 29)	*P*-value
Age(year)		31 ± 4.3	31.2 ± 5.5	31 ± 4.8	0.9^b^
Baseline BMI (kg/m^2^)		30.1 ± 3.1	30.5 ± 3.6	30.8 ± 3.2	0.7^b^
Pregnancy weight gain (kg)		15 ± 5.6	12.5 ± 4.4	13.9 ± 5.6	0.1^b^
Weight before pregnancy (kg)		68.9 ± 9.7	71.5 ± 12.3	70 ± 8.8	0.6^b^
Sun exposure of mothers (minutes/day)*	Baseline	10 (0–17.5)	10 (0–27)	10 (0–15)	0.7^c^
	Week 12	0 (0–5)	0 (0–0)	0 (0–0)	0.4^c^
Birth interval (year)*		4.8 (0–7.5)	3 (0–8.2)	4 (0–7.5)	0.7^c^
Number of children (n)*		2 (1–2)	2 (1–2)	2 (1–2)	0.9^c^
Hemoglobin (gr/dl)*		11.8 (11.3–12.4)	11.6 (11.2–12.6)	12.1 (11–12.5)	0.8^c^
Anemia n (%)		16 (55.2)	19 (59.4)	13 (44.8)	0.5^d^
Abortion history n (%)		6 (20.6)	9 (28.1)	7 (24.1)	0.7^d^
Cesarean delivery n (%)		24 (82.7)	31) 96.8)	28 (96.5)	0.06^d^
Multiple pregnancy n (%)		0 (0)	1 (13.1)	0 (0)	0.4^d^
Use of multivitamin during pregnancy n (%)		29 (100)	32 (100)	29 (100)	–
Use of iron supplement after delivery n (%)		29 (100)	32 (100)	29 (100)	–
Duration of breast feeding (weeks)		12	12	12	–
Exclusive breast feeding n (%)		29 (100)	32 (100)	29 (100)	–
Usage of sunscreen n (%) Baseline	Always Sometimes Never/Rarely	12 (41.4) 6 (20.7) 11 (37.9)	14 (43.8) 3 (9.4) 15 (46.9)	10 (34.5) 1 (3.4) 18 (62.1)	0.1^d^
Week 12	Always Sometimes Never/Rarely	14 (48.3) 3 (10.3) 12 (41.4)	12 (37.5) 6 (18.8) 14 (43.8)	10 (34.5) 2 (6.9) 17 (58.6)	0.4^d^
Physical activity level n (%)^e^ Baseline	Low Moderate High	10 (34.4) 11 (37.9) 8 (27.5)	17 (53.1)10 (31.2) 5 (15.6)	15 (51.7) 8 (27.5) 6 (20.6)	0.5^d^
Week 12	Low Moderate High	6 (20.6) 7 (24.1) 16 (55.1)	10 (31.2) 6 (18.7) 16 (50)	7 (24.1) 3 (10.3) 9 (65.5)	0.5^d^

The results are described as mean ± standard deviation or number %

*The results are described as median ± interquartile range (Q1 and Q3)

^a^Three groups of 2000 IU vitamin D3/d (VD1), 4000 IU vitamin D3/d (VD2), and placebo (PL)

^b^One Way ANOVA Test

^c^k Independent Samples (Kruskal-Wallis H Test)

^d^Chi Square Test

^e^Low: score less than 600 MET minutes per week, moderate: score 600–3000 MET minutes and high: score more than 3000 MET minutes

**Table 2:** Dietary intakes of nursing mothers at the baseline and after 12 weeks of intervention

Daily intake	mmeasurement periods	PL^a^ (*n* = 29)	VD1^a^ (*n* = 32)	VD2^a^ (*n* = 29)	*P*-value
Energy (kcal)	Baseline	2750.6 ± 725.2	2415 ± 589.8	2458 ± 663.1	0.1^b^
	Week 12	2374 ± 1172	2270 ± 1110	2569 ± 875.5	0.5^b^
Carbohydrate (g)	Baseline	367.2 ± 94.5	328.4 ± 88.3	338.5 ± 100.2	0.2^b^
	Week 12	315.9 ± 156.6	300.6 ± 147.2	351.4 ± 131.6	0.3^b^
Carbohydrate (%)	Baseline	53.5 ± 6.7	54.4 ± 6.1	54.8 ± 5.6	0.7^b^
	Week 12	53.2 ± 5.2	53 ± 5.7	54.4 ± 5.5	0.6^b^
Protein (g)*	Baseline	90.4 (68.4–97.6)	75.4 (63.3–90)	72.8 (65.2–87.6)	0.06^c^
	Week 12	74.8 (58.4–86.9)	70.9 (53.5–88.9)	84.7 (60.9–95.9)	0.5^c^
Protein (%)	Baseline	13 ± 2.6	12.5 ± 3.5	12.6 ± 2.3	0.7^b^
	Week 12	12.3 ± 2.7	12.9 ± 3.7	12.2 ± 2.1	0.6^b^
Fat (g)*	Baseline	105 (81.4–120.8)	88.2 (67.5–107.7)	87 (71–111.2)	0.1^c^
	Week 12	95.2 (69.8–136)	88.9 (72.4–111.2)	93.6 (85.7–122.2)	0.5^c^
Fat (%)	Baseline	34.8 ± 5.4	33.7 ± 6.9	33.8 ± 5.5	0.7^b^
	Week 12	35.6 ± 5.1	35.2 ± 4	34.3 ± 5.4	0.6^b^
Saturated fatty acids (g)	Baseline	27.7 ± 9.3	22.3 ± 7.3	23 ± 6.8	0.02^b^
	Week 12	23.5 ± 12.2	22.9 ± 12.3	23.4 ± 8.3	0.9^b^
Cholesterol (mg)*	Baseline	299.6 (254.7–418.3)	245 (183.3–346.5)	265.4 (181.4–318.6)	0.1^c^
	Week 12	269 (120.1–408.5)	224.6 (148.1–308.1)	236.8 (150–319.3)	0.6^c^
Mono-unsaturated fatty acids (g)*	Baseline	37.9 (26.4–40.3)	28.1 (24.4–35.3)	29.2 (22.3–38.4)	0.1^c^
	Week 12	33.4 (21.7–46.2)	32 (20.5–39.9)	32.1 (25.3–43.9)	0.8^c^
Poly-unsaturated fatty acids (g)*	Baseline	32.2 (22.9–40.9)	27 (18.7–39.7)	24.9 (20.4–34)	0.2^c^
	Week 12	32.6 (19.7–41)	26.3 (18.8–35.2)	30.9 (24.2–36)	0.4^c^
Dietary fiber (g)*	Baseline	19.7 (13.4–24.7)	18.3 (14.4–21.4)	17.1 (14.7–22.3)	0.4^c^
	Week 12	16.4 (12.5–24)	18.6 (11.9–21.9)	20.2 (15.8–24)	0.4^c^
Calcium (mg)*	Baseline Week 12	927.1 (648.9–1063.3) 765 (483–972.5)	925.1 (694.8–1048.4) 675.5 (405.2–1019)	891.1 (686.5–1059.2) 725 (464–1029.5)	0.9^c^ 0.8^c^
Vitamin D (μg)*	Baseline Week 12	1.3 (1.1–2.3) 0.2 (0–2.4)	1.2 (0–2.3) 0.3 (0–2.3)	1.3 (0.2–2.3) 1.1 (0–2.4)	0.8^c^ 0.9^c^

The results are described as mean ± standard deviation * The results are described as median ± interquartile range (Q1 and Q3)

^a^ Three groups of 2000 IU vitamin D3/d (VD1), 4000 IU vitamin D3/d (VD2), and placebo (PL)

^b^One Way ANOVA Test

^c^k Independent Samples Kruskal-Wallis H Test

### Effect of vitamin D3 supplementation on serum concentrations of 25(OH) D, iPTH, calcium and phosphorus

Serum 25(OH) D concentrations were significantly increased in the VD2 group (mean change (MC) 12.3 ng/ml; 95% CI, 9.4/15.0, *p*-value =0.001), but decreased in the VD1 and PL groups. A significant difference was observed between VD2 and VD1 (*p*-value = 0.001) and PL (*p*-value = 0.001) groups in terms of serum 25(OH) D changes. Vitamin D supplements did not affect serum calcium, iPTH, or phosphate measurements (*p*-value = 0.6) (Table 3).

**Table 3:** Biochemical analysis of 25-hydroxy vitamin D (25(OH) D), iPTH, calcium and phosphor of nursing mothers at baseline and week 12

Serum concentrations	Baseline		Week 12	Mean change (95% Confidence interval)	*P*-value ^b^
	VD1^a^	25.1 ± 11.2	20 ± 9.3	−5.1 (− 9.2/− 0.7)	0.001 [0.001]
25(OH)D (ng/ml)	VD2 ^a, c^ PL^a^	16.5 ± 6.7 26.0 ± 7.6	28.8 ± 9.7 20.4 ± 6.8	12.3 (9.4/15.0) − 5.6 (− 9.06/− 2.1)	
Phosphor (mmol/I)	VD1	3.7 ± 0.5	3.6 ± 0.4	− 0.01 (− 0.2/0.2)	0.4 [0.4]
	VD2	3.5 ± 0.8	3.7 ± 0.6	0.2 (− 0.1/0.5)	
	PL	3.5 ± 0.5	3.8 ± 0.6	0.25 (− 0.07/0.6)	
Calcium (mmol/I)	VD1	8.6 ± 0.6	9.5 ± 0.4	0.9 (0.5/1.2)	0. 6 [0.6]
	VD2	8.7 ± 0.6	9.3 ± 0.6	0.6 (0.2/1.0)	
	PL	8.3 ± 0.8	9.1 ± 0.5	0.8 (0.3/1.2)	
Intact Parathyroid Hormone (pg/ml)	VD1	3 ± 1.1	4.6 ± 1	1.1 (0.4/1.8)	0.5 [0.4]
	VD2	3.6 ± 1.6	4.7 ± 1.4	1.6 (1.0/2.2)	
	PL	3.1 ± 1.3	4.7 ± 1.6	1.6 (0.6/2.5)	

Data on baseline and week 12 are presented as mean ± standard deviation

^a^ three groups of 2000 IU vitamin D3/d (VD1), 4000 IU vitamin D3/d(VD2), and placebo (PL)

^b^ *P*-value is reported based on two-way repeated measure ANOVA (mixed ANOVA) to assess time effect and time by treatment effect interactions on all outcome variables after adjustment for saturated fatty acids intake before intervention. Numbers in brackets represent unadjusted *P* values

^c^ Significant difference with placebo and VD1 groups. (Obtained from two-way mixed ANOVA with Bonferroni correction)

### Effect of vitamin D3 supplementation on body composition

FM (MC − 4.3 kg; 95% CI, − 7.0/− 1.1, *p*-value =0.005), BFP (MC -8.1; 95% CI, − 12.0/− 4.2, *p*-value =0.007) and FMI (MC -1.6; 95% CI, − 2.6/− 0.5, *p*-value =0.005) were significantly decreased in the VD2 group as compared with the VD1 and PL groups. After adjusting for intake of SFA and mean change of weight, the statistical difference of FMI (*p*-value = 0.006), FM (*p*-value = 0.007), and BFP (*p*-value = 0.007) in the three groups remained significant. A significant difference was found between VD2, VD1 (*p*-value = 0.02), and PL (*p*-value = 0.008) groups in FM changes. In addition, a significant difference was observed between VD2, VD1 (*p*-value = 0.05), and PL (*p*-value = 0.008) groups in BFP changes. Furthermore, FMI changes were different between VD2 and VD1 (*p*-value = 0.02) and VD2 and PL groups (*p*-value = 0.008). Vitamin D3 supplementation showed no significant effect on body weight, BMI, WC, FFM, skeletal muscle mass, and RFMI (Table 4).

**Table 4:** Comparison of body composition measures at baseline and week 12 among nursing mothers

variable		Baseline	w	Mean change (95% Confidence interval)	*P*-value ^b^
Weight (kg)	VD1^a^	79.8 ± 11.8	73 ± 11.5	− 6.7 (− 9.1/− 4.5)	0.7 [0.7]
	VD2^a,C^	79.9 ± 10.6	73.7 ± 10	− 6.2 (− 8.3/− 4.2)	
	PL^a^	78.2 ± 8.4	72.6 ± 8.5	− 5.6 (− 6.7/− 4.5)	
Body Mass Index (kg m^2^)	VD1	30.5 ± 3.6	27.8 ± 4.7	− 2.7 (− 4.7/− 0.7)	0.6 [0.6]
	VD2	30.8 ± 3.2	28.8 ± 3.4	− 2.0 (− 4.0/− 0.01)	
	PL	30.1 ± 3.1	28.6 ± 4.1	− 1.5 (− 3.3/0.4)	
Waist Circumference (cm)	VD1	90.4 ± 8.3	91.7 ± 11.9	1.3 (− 3.9/6.5)	0.6 [0.8]
	VD2	91.7 ± 6.2	93.7 ± 3.2	2.0 (− 1.0/4.9)	
	PL	90.2 ± 6.8	93.1 ± 3.8	3.1 (0.5/5.7)	
Relative Fat Mass Index (m)	VD1	64.7 ± 0.9	64.6 ± 1.5	− 0.1 (− 0.8/0.5)	0.7 [0.8]
	VD2	64.5 ± 0.7	64.3 ± 0.4	− 0.2 (− 0.6/0.1)	
	PL	64.7 ± 0.8	64.3 ± 0.6	− 0.4 (− 0.7/− 0.06)	
Fat Free Mass (kg)	VD1	51.1 ± 6.04	45.6 ± 6.9	− 5.6 (− 8.8/− 2.3)	0.7 [0.7]
	VD2	49.6 ± 5.9	44.5 ± 4.1	− 5.0 (− 7.9/− 2.3)	
	PL	50.2 ± 5.9	43.5 ± 6	− 6.7 (− 9.9/− 3.5)	
Skeletal Muscle Mass (kg)	VD1	27.9 ± 3.5	24.2 ± 6.09	− 3.7 (− 6.1/− 1. 2)	0.7 [0.7]
	VD2	27.1 ± 3.5	24.5 ± 2.6	− 2.6 (− 4.4/− 0.9)	
	PL	27.5 ± 3.4	23.9 ± 3.8	−3.6 (− 5.5/− 1.6)	
Fat Mass (kg)	VD1	29.3 ± 6.1	30.3 ± 5.6	1.0 (− 1.9/3.9)	0.007 [0.005]
	VD2^c^	31.5 ± 6.7	27.2 ± 4.6	−4.3 (− 7.0 /− 1.1)	
	PL	27.8 ± 5.6	29.9 ± 6.9	2.1 (− 1.3/5.2)	
Percentage Of Body Fat	VD1	40.3 ± 6.9	38.7 ± 8.6	− 1.6 (− 5.5/2.4)	0.007 [0.007]
	VD2^c^	42.7 ± 7.2	34.6 ± 6.7	−8.1 (− 12.0/− 4.2)	
	PL	38.2 ± 5.5	38.7 ± 9.5	0.4 (− 3.4/4.3)	
Fat Mass Index (kg/m^2^)	VD1	11.2 ± 2.1	11.6 ± 2.1	0.4 (− 0.6/1.5)	0.006 [0.005]
	VD2^c^	12.1 ± 2.4	10.5 ± 1.8	− 1.6 (− 2.6/− 0.5)	
	PL	10.8 ± 2.4	11.6 ± 2.8	0.7 (− 0.3/1.9)	

Data on baseline and week 12 are presented as mean ± standard deviation

^a^ Three groups of 2000 IU vitamin D3/d (VD1), 4000 IU vitamin D3/d (VD2), and placebo (PL)

^b^ *P*-value is reported based on two-way repeated measure ANOVA (mixed ANOVA) to assess time effect and time by treatment effect interactions on all outcome variables after adjustment for mean change of weight (except for weight and body mass index) and saturated fatty acids intake before intervention. Numbers in brackets represent unadjusted *P* values

^C^ Significant difference with placebo and VD1 groups. (Obtained from two-way mixed ANOVA with Bonferroni correction)

### The relationship between stratified baseline and achieved serum 25(OH) D status and mean changes of body composition measures

More than 50% of participants had vitamin D deficiency at the study’s baseline. However, after 12 weeks of vitamin D supplementation, 70.5% of women who achieved sufficient serum 25(OH) D, of whom 41.9% were in the 2000 IU and 58.1% in the 4000 IU group. Mothers with baseline insufficient serum 25(OH) D (< 20 ng/ml) showed higher reduction in BMI, WC, RFMI, FM, PBF, and FMI. In addition, mothers who achieved sufficient serum 25(OH) D (> = 20 ng/ml) showed a higher reduction in weight, BMI, FM, PBF, and FMI. However, these differences were not statistically significant except for FMI (*p*-value = 0.04) and FM, which was close to significant (*p* = 0.055) (Table 5).

**Table 5:** Mean changes in body composition measures by stratified baseline and achieved serum 25- hydroxy vitamin D (25(OH) D) status ^a^

variable	Baseline serum 25(OH) D (ng/ml)		*p*-value ^b^	Achieved serum 25(OH) D (ng/ml)		*p-*value ^b^
	< 20 ng/ml	> = 20 ng/ml		< 20 ng/ml	> = 20 ng/ml	
*n* (%)	31 (50.8)	30 (49.2)		18 (29.5)	43 (70.5)	
	Mean change, mean ± SD			Mean change, mean ± SD		
Weight	−5.0 (5.6)	−8.0 (6.2)	0.06	−5.0 (5.4)	−7.1 (6.2)	0.2
BMI	−2.7 (5.5)	− 2.0 (5.3)	0.6	−1.6 (6.0)	− 2.7 (5.1)	0.4
Waist Circumference	2.4 (7.9)	0.7 (14.7)	0.5	3.5 (9.8)	0.8 (12.4)	0.4
Relative Fat Mass Index	− 0.3 (0.9)	−0.1 (1.9)	0.5	−0.4 (1.2)	− 0.1 (1.6)	0.4
Fat Free Mass	−6.2 (7.8)	−4.5 (8.6)	0.4	−6.7 (8.1)	−4.8 (8.3)	0.4
Skeletal Muscle Mass	−3.2 (4.7)	− 3.1 (6.8)	0.9	−2.5 (5.6)	− 3.5.(5.9)	0.5
Fat Mass	−3.4 (7.1)	0.5 (8.7)	0.055	0.3 (8.6)	−2.2 (7.9)	0.2
Percentage Of Body Fat	−6.4 (9.8)	−2.9 (12.3)	0.2	−1.4 (10.6)	−6.0 (11.2)	0.1
Fat Mass Index	−1.3 (2.8)	0.2 (3.2)	0.04	0.09 (3.2)	−0.8 (3.0)	0.3

^a^ The results are reported for two groups of 2000 IU vitamin D3/d (VD1) and 4000 IU vitamin D3/d (VD2) (*n* = 61)

^b^ Independent t-student test

## Discussion

Supplementation with 4000 IU vitamin D3 decreased FM, BFP, and FMI in nursing mothers with overweight or obese as compared with 2000 IU and placebo. Nevertheless, vitamin D3 supplementation did not significantly affect body weight, BMI, WC, FFM, skeletal muscle mass, and RFMI.

Only one previous study evaluated the effect of vitamin D supplementation on weight status in nursing mothers [25], which showed no effect of 1200 IU/d vitamin D on body composition. The results of a meta-analysis reported that by decreasing BMI and WC, vitamin D3 supplementation had a desirable effect on weight loss. However, the adequate dosages and supplementation duration are unclear [32]. In addition, no data were provided on other measures of body composition, such as FM and BFP. Another meta-analysis reported vitamin D supplementation did not affect BFP [33]. Since, the effect of vitamin D supplementation on weight loss is influenced by a large number of factors [34]. The results of clinical trials investigating the effect of vitamin D supplementation on weight loss in subjects with overweight or obese are inconclusive [35–37]. The study population is one of the primary causes of the disparity between our clinical trial outcomes and those of others. A systematic review study reported that most clinical trials with the null effect of vitamin D on health were performed in populations without vitamin D deficiency [38]. So possible beneficial effects from vitamin D supplementation cannot be excluded. Besides, in most clinical trials, a combined treatment of vitamin D and other alternatives was used [21, 35, 39].

At the end of the present study, the mean serum 25(OH) D in 2000 IU and placebo groups was decreased. A decrease in serum 25(OH) D concentrations during lactation has been shown [13]. Postpartum deterioration of 25(OH) D status might be explained by less frequent multivitamin intake during lactation [14, 40]. Therefore, a decrease in serum 25(OH) D concentrations in the placebo group was expected. Since each 400 IU/d of 25(OH) D increases serum 25(OH) D concentrations by 2.8 ng/ml in individuals with a normal BMI [41], it was expected that vitamin D3 supplementation at the dose of 2000 IU/d also increased maternal serum 25(OH) D concentrations similar to other studies [42, 43]. There are probably several reasons for this finding. First, an increase in serum 25(OH) D concentrations after vitamin D supplementation has been reported to be 30% lower in obese individuals compared with non-obese ones [44]. Therefore, the Society of Endocrine Guideline recommends that adults who suffer from overweight or obesity need 2 to 3 times more vitamin D supplementation (6000–10,000 IU/d) to treat and prevent vitamin D deficiency [8, 9]. Second, the physiological condition of the mothers during breastfeeding is very important. Approximately 20% of the 25 (OH) D in mother’s milk is transferred daily to the infant through breast milk [45].

In the present study, as in the only similar previous study [42], the dose of 4000 IU/d seems still too low to fully replenish vitamin D insufficiency in all participants. As previously explained, this is probably because the response of serum concentration of 25(OH) D to vitamin D supplementation is low during lactation due to the increase in the body’s need for maintaining bone mass [45], the transfer of 25(OH) D to milk and also the presence of overweight or obesity in the participants [35].

Mothers who achieved sufficient serum 25(OH) D showed a higher reduction in body composition measures. Similar results have been reported in other studies [34, 46]. However, these differences were not statistically significant except for FMI and FM, which were close to significant. This might be related to insufficient sample size in each serum category 25(OH) D. There are various mechanisms by which vitamin D has important roles in fat tissue metabolism [47]. Cholecalciferol supports intestinal calcium absorption, which can contribute to weight loss [39]. In addition, 25(OH) D prevents the rise in PTH secretion, which is associated with a reduction in lipolysis [48]. 25(OH) D also increases the expression of the peroxisome proliferator-activated receptor gamma (PPAR-γ) gene, which improves fatty acid metabolism [49].

Our study had several strengths and limitations. This was the first study investigating the effect of supplementation with vitamin D3 at either 2000 IU/day or 4000 IU/day on body composition and serum 25(OH) D concentration in nursing mothers with overweight or obesity. Another strength was that this was a randomized, double-blind, placebo-controlled clinical trial with an appropriate sample size for determining a reasonable effect size. The primary limitation of our study was that we could not assess body composition by Dual-Energy X-Ray Absorptiometry (DEXA) as a gold standard methodology. However, BIA is a validated and reliable method to assess body composition [24]. The second limitation was that liver fat or separate visceral and subcutaneous body fat was not determined in participants. The third potential limitation was the short duration of the intervention. The fourth limitation was the lack of measuring lipid profile, blood glucose and insulin sensitivity.

In conclusion, vitamin D3 supplementation at a dose of 4000 IU/d in nursing mothers with overweight or obesity improved serum 25(OH) D concentration, which had a beneficial effect on FM, BFP, and FMI. However, future long-term studies with different doses are required to confirm the results and determine the impact of vitamin D3 supplementation on liver fat and separate visceral and subcutaneous body fat in nursing mothers with overweight or obesity.

## Data Availability

The datasets generated during the current study are available via the corresponding author (GS) on reasonable request.

## Abbreviations

ANOVA: One-way analysis of variance
BFP: Body fat percentage
BIA: Body impedance analyses
BMI: Body mass index
DXA: Dual X-ray absorptiometry
ECL: Electro chemiluminescence
ELISA: Enzyme-linked immune sorbent assay
FMI: Fat mass index
FM: Fat mass
FFM: Fat free mass
ITT: Intention-to-treat
MET: Metabolic equivalents
MC: Mean change
PTH: Parathyroid hormone
RFMI: Relative fat mass index
SD: Standard deviation
SFA: Saturated fatty acids
VD1: Group of vitamin D3 supplementation (2000 IU/d)
VD2: Group of vitamin D3 supplementation (4000 IU/d)
WC: Waist circumference
25(OH) D: 25-Hydroxy vitamin D
